# Metabolic and morphological alterations induced by proteolysis-inducing factor from Walker tumour-bearing rats in C_2_C_12 _myotubes

**DOI:** 10.1186/1471-2407-8-24

**Published:** 2008-01-28

**Authors:** Claudia L Yano, Gislaine Ventrucci, William N Field, Michael J Tisdale, Maria Cristina C Gomes-Marcondes

**Affiliations:** 1 Departamento de Fisiologia e Biofísica, Instituto de Biologia, Universidade Estadual de Campinas (UNICAMP), CP 6109, 13083-970, Campinas, São Paulo, Brazil; 2 Cancer Research Laboratory, Pharmaceutical Sciences Research Institute, Aston University, Birmingham, B4 7ET, UK

## Abstract

**Background:**

Patients with advanced cancer suffer from cachexia, which is characterised by a marked weight loss, and is invariably associated with the presence of tumoral and humoral factors which are mainly responsible for the depletion of fat stores and muscular tissue.

**Methods:**

In this work, we used cytotoxicity and enzymatic assays and morphological analysis to examine the effects of a proteolysis-inducing factor (PIF)-like molecule purified from ascitic fluid of Walker tumour-bearing rats (WF), which has been suggested to be responsible for muscle atrophy, on cultured C_2_C_12 _muscle cells.

**Results:**

WF decreased the viability of C_2_C_12 _myotubes, especially at concentrations of 20–25 μg.mL^-1^. There was an increase in the content of the pro-oxidant malondialdehyde, and a decrease in antioxidant enzyme activity. Myotubes protein synthesis decreased and protein degradation increased together with an enhanced in the chymotrypsin-like enzyme activity, a measure of functional proteasome activity, after treatment with WF. Morphological alterations such as cell retraction and the presence of numerous cells in suspension were observed, particularly at high WF concentrations.

**Conclusion:**

These results indicate that WF has similar effects to those of proteolysis-inducing factor, but is less potent than the latter. Further studies are required to determine the precise role of WF in this experimental model.

## Background

The most common manifestation of advanced malignant disease is the development of cancer cachexia, which is a strong independent cause of mortality in this disease [1]. The abnormalities associated with cancer cachexia include anorexia, loss of body weight and muscle mass, as well as alterations in carbohydrate, lipid, and protein metabolism [2,3].

Atrophy of skeletal muscle results from increased protein catabolism (hypercatabolism) and decreased protein synthesis (hypoanabolism), both of which may occur simultaneously and result in intense muscular atrophy [4,5]. Cytokines, particularly TNF-α, IL-6, and interferon-γ, have been suggested to be responsible for the metabolic changes associated with tissue loss in cancer wasting [6-8]. In addition to humoral factors, tumour-derived molecules have also been proposed as mediators of cancer cachexia. Todorov et al. [9] purified and characterised a 24 kDa sulphated glycoprotein from the cachexia-inducing MAC16 tumour and similar material was also isolated from the urine of cachectic patients [9]. When this material was purified and injected into mice it produced a profound decrease in body weight, which was entirely due to loss of lean body mass [10]. Atrophy of skeletal muscle was due to a depression in protein synthesis (by 50%) and an increase in protein degradation (by 50%) [5]. The material was named proteolysis-inducing factor (PIF), because of its ability to directly induce protein loss in murine myotubes and isolated muscle preparations, and because this was the name given to an unidentified factor in human serum, which was capable of inducing proteolysis in isolated muscle preparations [11]. PIF induces protein degradation in skeletal muscle by induction of the ubiquitin-proteasome proteolytic pathway, which is considered to be the major mechanism by which myotubular proteins are degraded in skeletal muscle [12]. Recent results [13] show that PIF inhibits protein synthesis and induces protein degradation in skeletal muscle through a single step: the activation of the ds RNA-dependent protein kinase (PKR) [13]. Activation of PKR leads to phosphorylation of eukaryotic initiation factor 2 (eIF2) on the α-subunit, which inhibits translation initiation by competing with the guanine nucleotide exchange factor (eIF-2B) for the exchange of GDP for GTP on eIF2. Activation of PKR also leads to activation of the transcription factor NF-κB, which is responsible for the increased expression of major components of the ubiquitin-proteasome pathway. PIF may act alone or together with host or tumour derived cytokines to produce a cachectic state [14]. Both biochemical [15] and histological analysis [16] support the notion that tumours are the source of PIF.

Tumour growth induces marked changes in the oxidative metabolism of distant tumour-free tissues and organs of the host [17]. These changes include an increase in the level of pro-oxidant compounds and a reduction in the activities of antioxidant enzymes in extra-tumoral tissues [18]. A better understanding of the role of cytokines and tumour factors [19], in the molecular mechanisms of protein wasting in skeletal muscle is essential for the design of therapeutic strategies in the near future. The Walker-256 tumour has been extensively used as an experimental model to induce cachexia in rats [20], but there have been no studies on whether the tumour and/or host cells produce a factor similar to PIF. In this study, we investigated the metabolic effects of 'Walker factor' (WF) purified from ascitic fluid from rats bearing the Walker 256 tumour, and its relationship to PIF.

## Methods

### Animals and tumour implantation

Adult male Wistar rats were obtained from the Multidisciplinary Centre for Biological Investigation (CEMIB) at UNICAMP. The rats were maintained on a 12 h light/dark cycle at 23°C and fed standard rodent chow. All of the experimental protocols involving animals were reviewed and approved by the Institutional Committee for Ethics in Animal Experimentation (CEEA/IB/UNICAMP, protocol no. 342-1). Walker 256 cells were cultured intraperitoneally and the ascitic fluid was obtained from the peritoneal cavity as described by Gomes-Marcondes et al. [21].

### "Walker factor" purification

The ascitic fluid obtained from Walker 256 tumour-bearing rats was centrifuged to remove the tumour cells and the high molecular weight proteins of the supernatant were precipitated with 40% ammonium sulphate and centrifuged (3000 × g). The supernatant was dialysed with PBS to remove the ammonium sulphate and then concentrated by filtration through an Amicon Ultra centrifugal filter, 10,000 MW cut off (Millipore Corporation, USA) prior to concentration by affinity chromatography [9]. The total protein was measured by a colorimetric method [22]. The 24 kDa protein present in the protein concentrate from Walker tumour ascitic fluid (WF) was resolved by 12% SDS-polyacrylamide gel electrophoresis, stained by Amido Black (1%) and further quantified by western blotting using an antibody to PIF (purified by Cancer Research Laboratory, Aston University, Birmingham, UK). No protease activity was found in the Walker factor after a zymography assay; using electrophoresis in the presence of SDS in 10% polyacrylamide gels and 1 mg/ml gelatin, the Walker factor protein was renatured by exchanging SDS and incubated for 22 hours in Tris-HCl buffer (50 mmol/L), followed by Coomassie Blue staining and densitometric analysis.

### Cell culture

C_2_C_12 _myoblasts, obtained from the Cancer Research Laboratory, Aston University, Birmingham, UK, were grown in 75 cm^2 ^tissue culture flasks (Corning, NY) at 37°C in a humidified 5% CO_2 _atmosphere in medium (DMEM; Sigma, St. Louis, MO) supplemented with 10% foetal calf serum (FCS; Sigma) and 1% penicillin/streptomycin (Sigma). All experiments were initiated using cells grown to 90–100% confluence. To induce differentiation, the growth medium was replaced by medium supplemented with 2% horse serum. 'Walker factor' was incubated with the cells for 24, 48 and 72 h at final concentrations of 3, 5, 10, 15, 20 and 25 μg.mL^-1^.

### Cytotoxicity assays

The cell viability of control and WF-treated C_2_C_12 _myotube cultures was assessed based on MTT reduction, neutral red uptake (NRU) and nucleic acid content (NAC). The MTT assay is a sensitive, quantitative colorimetric assay that measures cell viability based on the capacity of mitochondrial succinyl dehydrogenase in living cells to convert the yellow substrate (3-(4,5-dimethylthiozol-2-yl)-2,5-diphenyltetrazolium bromide, MTT; Sigma) into a dark blue formazan product. After incubation with a 0.01% solution of MTT for 10 min at 37°C, the medium was removed and the formazan solubilised in ethanol. The plate was shaken for 30 min and the absorbance was measured at 570 nm [23]. The NRU assay is a cell viability test based on the incorporation of dye into the lysosomes of viable cells after incubation with the test agents. After removing the medium from the plates, a 0.05% solution of neutral red was added to each well followed by incubation for 3 h at 37°C. The cells were then washed with phosphate-buffered saline containing calcium (PBS-Ca^2+^), followed by the addition of a solution of 1% glacial acetic acid in 50% ethanol to fix the cells and extract the neutral red incorporated into the lysosomes. The plates were shaken for 20 min and the absorbance was measured at 540 nm [24]. The NAC was determined in the same plates used for the NRU assay. The cell monolayer was solubilised with 0.5 N NaOH at 37°C for 1 h after which the absorbance was measured at 260 nm and expressed as a percentage of the control [25].

### Analytical methods

After 24, 48 and 72 h of treatment, the cells were washed in cold PBS to slow biochemical reactions, and then collected in homogenising buffer (HB) (20 mM Tris, 1 mM DTT, 2 mM ATP and 5 mM MgCl_2_, pH 7.2), and centrifuged at 10,000 rpm for 15 min at 4°C. Aliquots of the homogenate supernatant were analysed for glutathione-S-transferase (GST) activity based on the conjugation of 1-chloro-2,4-dinitrobenzene (CDNB; Sigma) with glutathione, and the activity was expressed as nmol.μg protein^-1^.min^-1^, using an extinction coefficient of 9.6, as described by Habig et al [26]. The lipid peroxidation product malondialdehyde (MDA) was determined by incubation with MPO (n-methyl-2-phenylindole; Sigma), followed by measurement of the absorbance at 590 nm, and the results were expressed as nmol.μg protein^-1 ^[27]. The chymotrypsin-like activity was determined by incubating 10 μL of the homogenate supernatant with 100 μL of the fluorogenic substrate succinyl-Leu-Leu-Val-Try-7-amino-4-methylcoumarin (Suc LLVY-AMC) (0.167 μg.μL^-1 ^in Tris-HCl, pH 7.4), followed by measurement of the fluorescence (excitation: 360 nm, emission: 460 nm), with the results being expressed as fluorescence.μg protein^-1^.

### Measurement of total protein synthesis and degradation

Differentiated myotubes used for protein synthesis were grown to confluence then treated with WF (3, 5, 10, 15, 20 and 25 μg.mL-1) for 24, 48 and 72 h. After each treatment period, the cells were analysed for protein synthesis using procedures adapted from White et al [28] with the following modifications: the myotubes were labelled with L-[2,6^3^H] phenylalanine (0.67 μCi.mmol-1 in DMEM added with cold phenylalanine (2 mmol.L-1) for 1 h. Subsequently, the cells were washed twice with 10% TCA, and then were removed from the plates and centrifuged. The cellular pellet was prepared by protein precipitation with 10% TCA, which was removed, and 0.5 M NaOH was added, together with 0.1% Triton, and the incubation was carried out for 2 h at 37°C. The protein bound radioactivity was determined in 6 mL scintillation fluid, and expressed as CPM.μg protein^-1^.

The myotubes were analysed for protein degradation using procedures from Gomes-Marcondes et al [12]. Differentiated myotubes grown in six-well plates were labelled with L-[2,6^3^H] phenylalanine (0.67 μCi.mmol^-1 ^in DMEM added with cold phenylalanine 2 mmol.L^-1^) in 2 mL DMEM containing 2% horse serum. After 24 h, the myotubes were washed three times in PBS, followed by the addition of fresh DMEM without phenol red, and then the cells were subsequently treated with WF (3, 5, 10, 15, 20 and 25 μg.mL^-1^) for 24, 48 and 72 h. The protein degradation was determined measuring the radioactivity of the cell supernatant released into the medium using a Beckmam LS 6000TA liquid scintillation counter, and expressed as CPM per μg of cellular protein.

### Light and scanning electron (SEM) microscopy

C_2_C_12 _myotubes were grown on coverslips and treated with different concentrations of WF. After 24 h of treatment, the cells were analysed by light microscopy (Leica Instruments). Some cells were fixed in 2.5% paraformaldehyde/glutaraldehyde (Sigma) in 0.1 M PBS, pH 7.4, and then washed in PBS followed by post-fixation with 1% osmium tetroxide (Sigma) and dehydration in a graded ethanol series. The cells were subsequently dried to the critical point (CPDO030 – Balzers, BAL-TEC AG, Wiesbaden, Germany) and gold sputtered (SCD050 – Balzers) before being analysed in a scanning electron microscope (JSM-5800LV, JEOL, Peabody, MA, USA) operated at 1 kV.

### Statistical analysis

The results were expressed as the mean ± SEM. One-way ANOVA followed by Bonferroni's test was used to compare the WF-treated groups with the controls [29]. A value of P < 0.05 indicated significance.

## Results

**Figure 1:**
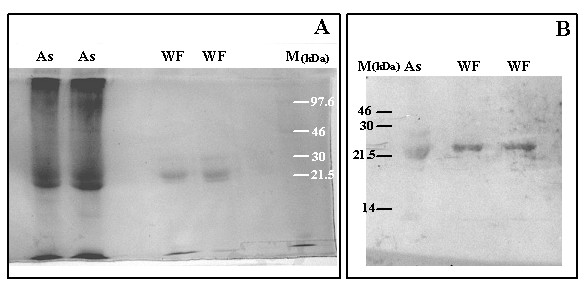
Detection of WF after the purification of ascitic fluid from Walker tumour-bearing rats. A – pure ascitic fluid (As) and purified WF were loaded (10 μg protein) in 12% SDS-polyacrylamide gel followed by 1% Amido Black staining. B – samples of pure ascitic fluid (As) and purified WF were resolved by SDS-PAGE on 12% gels followed by Western blotting; the WF expression were analysed by probing 0.45 mm enhanced chemical luminescence (ECL) nylon membranes with biotinylated anti-PIF antibody (diluted 1:150) followed by detection with the secondary antibody streptavidin horse radish HRP-labelled (dilution 1:1000). Images of the stained and Western blot gels were captured (FTI 500 Image Master VDS, Pharmacia Biotech). Legend: As – pure ascitic fluid, WF – Walker factor obtained by purification and concentration of ascitic fluid, M – marker (220 to 14 kDa).

The present work shows that it is possible to purify and detect the presence of a factor, named here as the "Walker Factor"(WF), isolated from ascitic fluid of Walker 256 tumour-bearing rats. The WF appears immunologically identical with PIF (Figure 1), and has the same molecular weight (24 kDa). The WF was purified by affinity chromatography using anti-PIF antibody and represented around 1.2% of the total protein, after the concentration. Further the WF was resolved as a single band by SDS-PAGE electrophoresis (Figure 1A) and further detected by Western blotting using a murine antibody to PIF which detects the oligosaccharide chains on the glycoprotein (Figure 1B).

**Figure 2:**
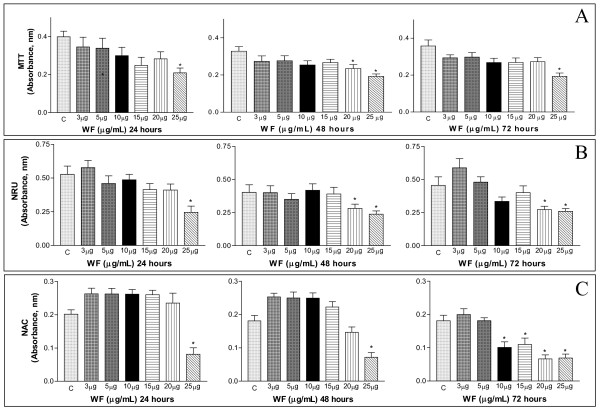
The influence of WF on C_2_C_12 _cell viability assessed by the MTT reduction (A, absorbance nm), NRU (B, absorbance nm) and NAC (C, absorbance nm) assays. C_2_C_12 _myotubes were treated with WF (3, 5, 10, 15, 20 and 25 μg.mL-1) for 24 h, 48 h and 72 h.

To determine whether the WF could have effects similar to PIF, we used the MTT, NRU and NAC assays to examine the effects of WF on C_2_C_12 _myotubes after treatment with various concentrations of WF (up to 25 μg.mL^-1^) for 24, 48 and 72 h. Although the WF has a molecular weight similar to PIF [9,30] the doses required to produce detectable physiological effects are higher than those for PIF [12,13]. The present results showed that WF was cytotoxic to cultured C_2_C_12 _myotubes at higher concentrations than PIF. The cell viability decreased after 24 h incubation with a WF concentration of 25 μg.mL^-1^; after 48 h and 72 h, WF affected the cell viability mainly at concentrations of 20–25 μg.mL^-1 ^(Figure 2A). The cellular lysosomal response was also significantly affected at high WF concentrations (20 and 25 μg.mL^-1^), particularly after 48 h and 72 h (Figure 2B). The NAC decreased in cells treated with 10 and 25 μg of WF.mL^-1^, especially after 72 h (Figure 2C), a finding corroborated by the MTT and NRU assays.

**Figure 3:**
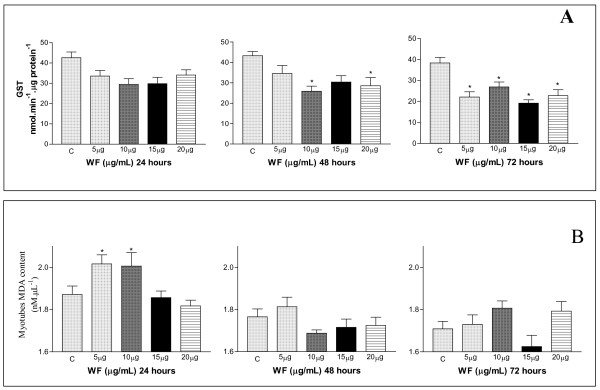
The influence of WF on the glutathione-S-transferase activity (A, nmol.min^-1^.μg protein^-1^) and malondialdehyde content (B, μg.μL^-1^) of C_2_C_12 _myotubes treated with WF (3, 5, 10, 15, 20 and 25 μg.mL^-1^) for 24 h, 48 h and 72 h.

Treatment with low concentrations of WF (5–10 μg.mL^-1^) for 24 h reduced the GST activity of C_2_C_12 _cells, and this decrease was particularly pronounced after 72 h (Figure 3A) In contrast, there was a significant increase in the MDA levels at WF concentrations of 5 μg.mL^-1 ^and 10 μg.mL^-1 ^(2.0 and 2.8 times higher than in control cells, respectively) (Figure 3B).

**Figure 4:**
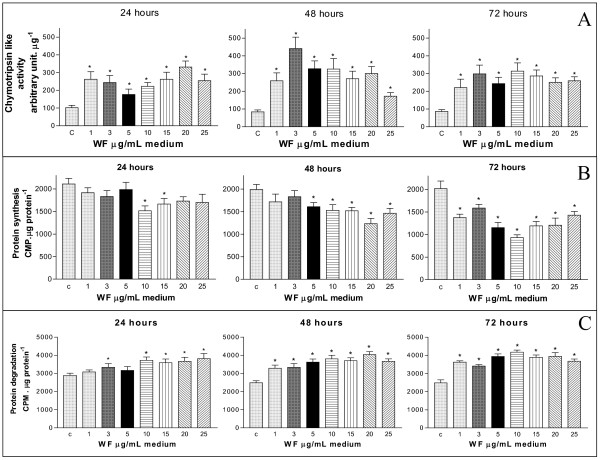
The influence of WF on the chymotrypsin-like activity (A, % arbitrary intensity of the control μg protein^-1^), protein synthesis (B, CPM.μg cellular protein^-1^) and protein degradation (C, CPM.μg protein^-1^) in C_2_C_12 _myotubes incubated with WF (3, 5, 10, 15, 20 and 25 μg.mL^-1^) for 24 h, 48 h and 72 h.

All concentrations of WF (1 to 25 μg.mL^-1^) increased the 'chymotrypsin-like' enzyme activity of C_2_C_12 _myotubes after 24 h, and a further increase was observed especially in 3, 5 and 10 μg.mL^-1 ^after 48 h; the 'chymotrypsin-like' enzyme activity remained enhanced in all concentrations tested after 72 h, indicating enhanced cellular catabolism of proteins (Figure 4A). The chymotrypsin activity was suppressed when the C_2_C_12 _myotubes were treated with 10 μM lactacystin (a proteasome inhibitor) together with WF (data not shown). In contrast, WF (only at 10 and 15 μg.mL^-1^) reduced the protein synthesis in C_2_C_12 _myotubes after 24 h (Figure 4B), with a tendency to a decrease in protein synthesis at all concentrations. The protein synthesis inhibition was statistically significant and greater at all WF concentrations and with longer treatments (48 h and 72 h) (Figure 4B). The WF induced an enhancement in protein degradation in C_2_C_12 _myotubes, in 3, 10, 15. 20 and 25 μg.mL^-1^, after 24 h, and this was maintained up to 72 h treatment (Figure 4C).

**Figure 5:**
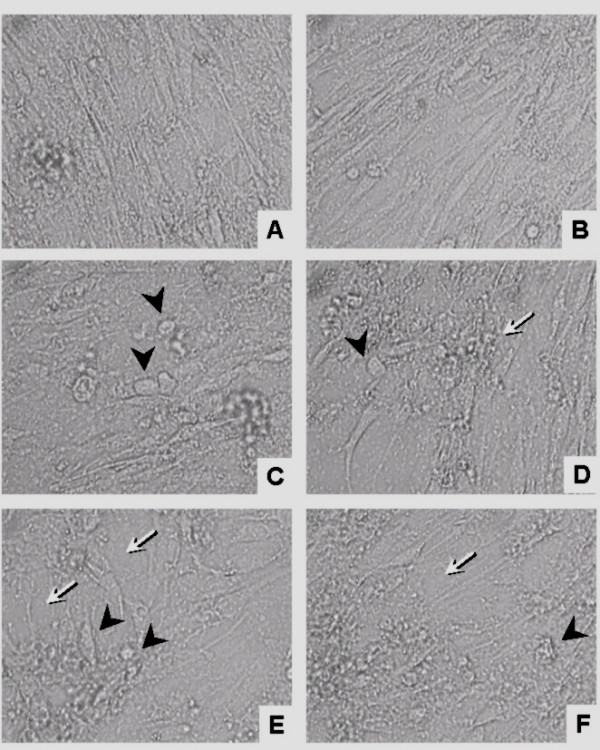
Light microscopy of C_2_C_12 _myotubes treated with WF (5, 10, 15, 20, 25 μg.mL^-1 ^for 24 h). A, in the absence of WF, C_2_C_12 _myotubes had a normal, elongated shape. B-F, C_2_C_12 _myotubes incubated for 24 h with 5 (B), 10 (C), 15 (D), 20 (E) and 25 (F) μg of WF.mL^-1^. The morphological analysis of WF-treated C_2_C_12 _cells revealed a loss of cell-to-cell contact and consequent detachment (white arrows), cell retraction and a change in cell shape (arrowheads). Lens 40×.

**Figure 6:**
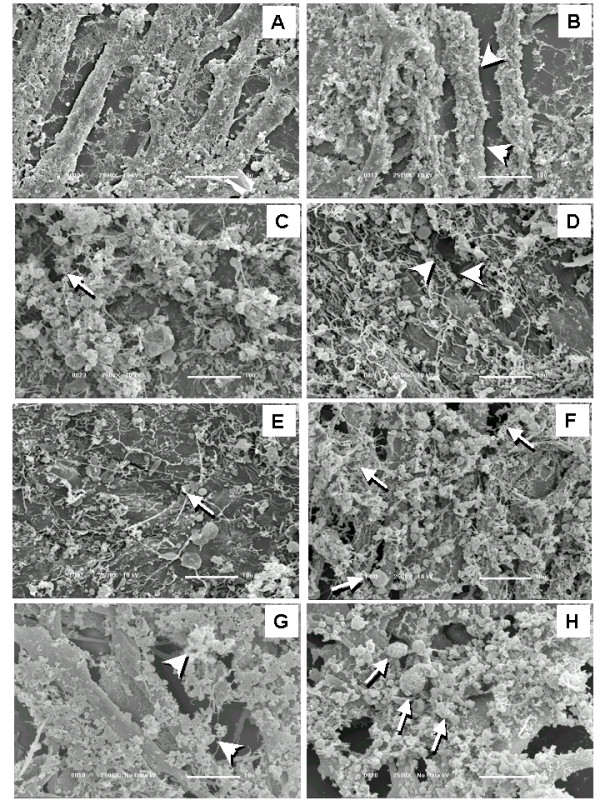
SEM of C_2_C_12 _myotubes treated with WF (5, 10, 15, 20, 25 μg.mL^-1 ^for 24 h). A, in the absence of WF, C_2_C_12 _myotubes had a normal, elongated shape. B-H, C_2_C_12 _myotubes incubated for 24 h with 1 (B), 3 (C), 5 (D), 10 (E), 15 (F), 20 (G) and 25 (H) μg of WF.mL^-1^. The morphological analysis of WF-treated C_2_C_12 _cells revealed a loss of cell-to-cell contact, with consequent detachment and damage to the collagen matrix (arrowheads), cell retraction, altered cell shape and vesicle formation (arrows in panel E, F and H). 2500×.

Light and scanning electron microscopy showed that WF produced morphological changes. Light microscopy showed that C_2_C_12 _myotubes grown in complete medium in the absence of WF had a normal, elongated shape (Figure 5A). However, after 24 h incubation with WF (5, 10, 15, 20 and 25 μg.mL^-1^), morphological changes such as a loss of cell-to-cell contact and subsequent detachment, cell retraction and an altered cell shape were observed (Figure 5B–E). The loss of cell-cell contact was particularly evident at a high concentration (25 μg.mL^-1^) of WF (Figure 5E). Morphological changes were also seen in SEM after incubation with 1, 3, 5, 10, 15, 20, and 25 μg of WF.mL^-1 ^for 24 h. Compared to untreated (control) cells (Figure 6A), treatment with WF resulted in numerous membrane vesicles and cell detachment, with cell retraction leading to changes in cell shape. There was also strong evidence of collagen matrix degradation (Figure 6B–E). All these confirmations together were seen especially at high concentrations of WF (Figure 6F and 6H). The morphological alterations induced by WF resulted in irreversible cell damage and wasting.

## Discussion

Cancer-cachexia may be defined as a severe state of malnutrition, characterised by anorexia, weight loss, muscle wasting, and anaemia. Although cachexia often involves a reduced food intake, it differs from starvation in that there is also evidence of metabolic changes in response to cytokines or humoral factors produced by the host [11] or by tumour cells [9] that act directly on skeletal muscle to reduce protein synthesis or increase proteolysis. The present work shows for the first time that a PIF-like molecule has been detected in the ascitic fluid from Walker tumour-bearing rat. This is the first report of a PIF-like molecule from a tumour-bearing rat, although it has previously been identified in tumours from both mice [9] and humans [15]. The WF has both immunological and molecular weight characteristics identical to PIF, although it appears to be less potent in inducing a biological response. PIF shows activity in the nM range [9,13] whereas the WF requires concentrations up to 1 μM for maximal biological effect. The reason for this difference is not known, but could be due to small differences in the composition of the oligosaccharide chains, which are essential for the biological activity of PIF [31].

Patients with cancer-induced weight loss show reduced protein synthesis in skeletal muscle [32] and increased protein degradation, with the latter resulting from an increased expression of the ubiquitin-proteasome proteolytic pathway, the major determinant of protein degradation. In agreement with this, skeletal muscle from cachectic cancer patients shows increased expression of mRNA for ubiquitin [33] and the proteasome subunits [34] with proteasome-mediated proteolytic activity increasing in parallel with ubiquitin expression [33]. As in cancer cachexia, there was a marked increase in chymotrypsin-like enzyme activity, the major catalytic activity of the 20S β proteasome catalytic subunit, [35], in myotubes after treatment with WF. Protein degradation was enhanced in C_2_C_12 _myotubes treated with WF, in parallel to an intense depression of protein synthesis, as observed in skeletal muscle of cachectic cancer patients.

The Walker-256 tumour is an experimental model of cancer-cachexia [20] and produces marked changes in the plasma levels of hormones and cytokines [36-39] including WF isolated from the ascitic fluid from tumour-bearing animals, as studied here. Although few studies have examined the humoral factors produced by Walker tumour-bearing rats, the present work observed for the first time that WF has comparable molecular weight and immunogenicity as PIF and has been shown to produce similar effects [9] but at higher concentrations. Although there have been no direct measurements of WF in tumour tissue, in analogy with other studies [15,16] it could be produced by the Walker-256 tumour cells and/or host tissue cells as in other study showed that the original PIF core-protein was also expressed in non-tumour cells assessed in patients with oesophageal carcinoma [40]. PIF is a complex sulphated glycoprotein with a short core peptide chain to which is attached both N- and O-linked oligosaccharide chains [31]. Over 80% of the molecule is carbohydrate. Although mRNA for the PIF core peptide is upregulated in both tumour and adjacent normal tissue in gastro-oesophageal malignancy, this does not relate to prognosis or cachexia [40]. This is because the biological activity of PIF is due to the complex oligosaccharide chains [31]. Thus post-translational modification of the PIF core peptide is a key step in determining the biological role, and evidence to date suggests that this can only be carried out by tumour tissue.

The humoral factors play an important role in the intense catabolism of carbohydrates, lipids and protein seen in cancer cachexia [36,41,42]. Vasopressin, prostaglandin E_2 _and TNF-α have been proposed to be involved in the development of cancer cachexia in Walker-256 tumour-bearing rats [35,36]. The infusion or injection of cytokines into animals decreases the protein content of skeletal muscle by increasing the rate of protein degradation and decreasing the rate of protein synthesis [43]. Similar responses were seen here using cultured C_2_C_12 _myotubes.

Tumour-bearing animals can present changes in the activities of oxidant and antioxidant enzymes that influence the antioxidant capacity of skeletal muscle and may lead to oxidative stress [12]. Previous studies have shown decreased catalase activity in the liver and kidneys of tumour-bearing rats [44,45]. In the plasma of cancer patients, there are also marked changes in the activities of antioxidant enzymes [46]. These changes have been attributed to an increase in TNF-α that reduces hepatic catalase activity [46]. Glutathione peroxidase and transferase are the major enzymes involved in protection against cells peroxidative damage [47]. In addition to a possible control by hormones and cytokines, reactive oxygen species (ROS) may also regulate the activity of antioxidant enzymes [48]. In the present study, WF, especially at high concentrations, was extremely cytotoxic, as shown by the decrease in NAC (which reflects a decrease in DNA content). Additionally, the reduced GST activity seen mainly after 48 h and 72 h of treatment indicated a loss of cellular protective mechanisms and increased in oxidative stress as expressed as malondialdehyde content 24 h after WF treatment, inducing a cumulative damage on myotubes. These results agree with those of Tang et al. [49], who demonstrated that metabolites of the lipoxygenase (12-LOX) pathway were involved in the survival of Walker cells by protecting against the increase in lipid peroxidation and the loss of glutathione-S-transferase activity.

Treatment with WF produced morphological changes in C_2_C_12 _myotubes that involved disruption of the collagen matrix and the loss of cellular adherence, events suggestive of physiological changes associated with programmed cell death (apoptosis). Although apoptosis has been widely studied in a variety of tissues in recent years, the importance of this phenomenon in skeletal muscle and heart in individuals with cancer has been relatively ignored. Nevertheless, apoptosis has an important role in specific cardiac pathologies, particularly in response to ischemia-perfusion in which ROS are formed. In skeletal muscle, very few studies have been done under specific physiological (e.g., exercise) and pathophysiological (e.g., dystrophies, denervation, myopathies) conditions. Skeletal muscle is unique in that it is multi-nucleated and can undergo individual myonuclear apoptosis as well as complete cell death [50]. Meanwhile, WF may affect the morphological integrity of the C_2_C_12 _myotubes, especially the matrix collagen by the activation of NF-κB which induces the stimulation of matrix metalloproteinases [51] in addition to the ubiquitin-proteasome pathway. PIF induces degradation of I-kappaBalpha and nuclear accumulation of NFκB in myotubes enhancing the protein degradation [52]. Therefore this fact could be involved in the degradation of the cell matrix and altering the normal cell activities, thereby leading to cell death. Further experiments are underway to determine how WF may cause this damage in C_2_C_12 _myotubes.

## Conclusion

Rapid neoplasic growth induces detrimental changes in the host as a result of carcass wasting. WF may produce its effects by direct and indirect mechanisms that involve the inhibition of protein synthesis and increase the protein degradation associated with an increase in oxidative stress in C_2_C_12 _myotubes, in a manner similar to the host tissue wastage seen in cancer patients.

## Abbreviations

CDNB – 1-chloro-2,4-dinitrobenzene; DMEM – Dulbecco's modified Eagle's medium; ECL – enhanced chemical luminescence FCS – foetal calf serum; GST – glutathione-S-transferase; HB – homogenising buffer; HRP – horse radish peroxide; IL6 – interleukin 6; MDA – malondialdehyde; MTT – 3-(4,5-dimethylthiozol-2-yl)-2,5-diphenyltetrazolium bromide; NAC – Nucleic acid content; NRU – neutral red uptake; PBS – phosphate buffered saline; PIF – proteolysis inducing factor; ROS – reactive oxygen species; SDS-PAGE – sodium dodecyl sulphate polyacrylamide gel electrophoresis; SUC-LLVY-AMC – succinyl-Leu-Leu-Val-Try-7-amino-4-methylcoumarin; TCA – tricloro acetic acid; TNFα – tumour necrosis factor; WF – Walker Factor.

## Competing interests

The author(s) declare that they have no competing interests.

## Authors' contributions

The author CLY, GV and WJF contributed to the data collection, analysis, data interpretation, and manuscript preparation. The author MJT participated in the design of the study and contributed to manuscript preparation. The author MCCGM conceived the study and its design and coordinated the works and manuscript preparation. All authors read and approved the final manuscript.

## Pre-publication history

The pre-publication history for this paper can be accessed here:


